# Desire versus Efficacy in Smokers’ Paradoxical Reactions to Pictorial Health Warnings for Cigarettes

**DOI:** 10.1371/journal.pone.0054937

**Published:** 2013-01-29

**Authors:** Daniel Romer, Ellen Peters, Andrew A. Strasser, Daniel Langleben

**Affiliations:** 1 Annenberg Public Policy Center, University of Pennsylvania, Philadelphia, Pennsylvania, United States of America; 2 Department of Psychology, Ohio State University, Columbus, Ohio, United States of America; 3 Center for Interdisciplinary Research on Nicotine Addiction, Department of Psychiatry, University of Pennsylvania, Philadelphia, Pennsylvania, United States of America; 4 Center for the Study of Addiction, Department of Psychiatry, University of Pennsylvania, Philadelphia, Pennsylvania, United States of America; University of Granada, Spain

## Abstract

Pictorial health warnings on cigarette packs create aversive emotional reactions to smoking and induce thoughts about quitting; however, contrary to models of health behavior change, they do not appear to alter intentions to quit smoking. We propose and test a novel model of intention to quit an addictive habit such as smoking (the efficacy-desire model) that can explain this paradoxical effect. At the core of the model is the prediction that self-efficacy and desire to quit an addictive habit are inversely related. We tested the model in an online experiment that randomly exposed smokers (N = 3297) to a cigarette pack with one of three increasing levels of warning intensity. The results supported the model’s prediction that despite the effects of warnings on aversion to smoking, intention to quit smoking is an inverted U-shape function of the smoker’s self-efficacy for quitting. In addition, smokers with greater (lesser) quit efficacy relative to smoking efficacy increase (decrease) intentions to quit. The findings show that previous failures to observe effects of pictorial warning labels on quit intentions can be explained by the contradictory individual differences that warnings produce. Thus, the model explains the paradoxical finding that quit intentions do not change at the population level, even though smokers recognize the implications of warnings. The model suggests that pictorial warnings are effective for smokers with stronger quit-efficacy beliefs and provides guidance for how cigarette warnings and tobacco control strategies can be designed to help smokers quit.

## Introduction

Cigarette smoking accounts for over 430,000 deaths annually in the U.S. [1] and is responsible for over 5 million fatalities per year worldwide [2]. Efforts to educate the public about the hazards of smoking have been ongoing since they were first identified [3]. These efforts along with restrictions on advertising and locations where people can smoke have steadily reduced the prevalence of smoking in the U.S. from a high of 42% in 1965 to about 20% in most recent surveys [4]. In addition, rates of initiation in adolescents have declined, thereby reducing the recruitment of new smokers to the population [4], [5]. Despite these successes, the rate of quitting smoking in recent years has declined and, although many try to quit, only about 5% are successful annually [6]. As a result of this and a growing population, there are almost as many smokers in the U.S. today as there were at the height of the epidemic in the 1960’s. Clearly, in order to continue reducing smoking prevalence, greater efforts will be needed to reach smokers who fail to quit.

One effort by the U. S. government to encourage quitting has been to place textual warnings about the hazards of smoking on the sides of cigarette packs. Although such warnings have been in place in the U.S. since 1965, they have not changed since 1984 and are easy to ignore [7], [8]. In an effort to increase the effectiveness of these warnings, recent legislation empowers the U. S. Food and Drug Administration (FDA) to impose larger pictorial warnings on the front and back of cigarette packs similar to those that were first introduced in Canada and elsewhere. Research indicates that these enhanced warnings not only draw the smoker’s attention but also succeed in creating aversive emotional reactions to the prospect of smoking [9], [10]. In addition, studies of the effects of introducing pictorial warnings in Australia and the UK indicate that they increase smokers’ *thoughts about* quitting [11], [12].

These findings have led researchers and policy makers to conclude that the warnings work, despite the lack of direct evidence that they increase quit rates [9]. Indeed, research conducted to evaluate immediate effects of pictorial warnings in the U.S. indicates that the warnings seldom change intentions to quit [13], [14]. A large FDA test of 36 different pictorial warning labels presented to two age groups of smokers (18–24 vs. 25+) revealed that out of 72 tests, only 6 increased intentions *to try to* quit [13]. A smaller replication with fewer warnings but larger sample sizes per condition found that, although pictorial warnings enhanced smokers’ aversion to smoking, they produced no overall effects on intentions to try to quit in the near future [14].

Intentions are important because they are critical precursors to behavior change [15]. Unless a smoker strongly intends to quit, it will be difficult, if not impossible, to overcome the cravings and withdrawal symptoms that maintain this addictive habit [16]. Indeed, models of health behavior change, such as Protection Motivation Theory (PMT) [17], the Health Belief Model (HBM) [18], and the Theory of Reasoned Action (TRA) [15], predict that intentions should increase as the perceived risks of smoking increase. Nevertheless, although pictorial warnings encourage smokers to *think about quitting*, the warnings do not appear to enhance the likelihood that the average smoker will actually try to quit. Thus, the failure of warnings to influence intentions poses a paradox for any theory that assumes that people act in their own best interests, especially when they recognize threats to those interests.

Recent neuroscience research provides insight into the paradoxical effects of warning labels. This research has identified two neuropsychological systems that influence the development of an addiction and that explain why smoking cessation is difficult. First, ingestion of nicotine, the addictive drug in tobacco, alters the mesocorticolimbic dopamine system that controls expectations of reward [19]. Over time, these expectations become conditioned to the act of smoking itself, thus making the person who smokes sensitive to any cues associated with the act and enhancing the desire to smoke when exposed to them [20], [21]. Second, repeated acts of smoking transfer control over the habit to dorsal striatal circuits that undermine prefrontal control [22], [23] and that turn the habit into a compulsion, leaving the smoker with reduced sense of control over the behavior [24], [25]. Although this description of the two systems is necessarily abbreviated, it is clear that these changes in the reward and control systems make it difficult for the addict to resist the pull of smoking cues and the craving elicited by them. Thus, despite the desire to quit that most smokers report [26], their perceived efficacy to do so is lacking. This conflict between desire and efficacy often leaves smokers without sufficient motivation and, hence, intention to quit the habit.

In view of the powerful neuropsychological processes identified by neuroscience research, we translated those insights into a behavioral decision making model that can account for the paradoxical finding that despite enhancing desires to quit, warnings do not appear to change intentions to do so. We first describe the model and then present a test of its major predictions.

### A Model of Intentions to Quit an Addictive Behavior such as Smoking

The efficacy-desire model (EDM) proposes that the intention to quit smoking (*Iq*) is a function of the difference between the motivation to smoke (*Ms*) and the motivation to quit (*Mq*):
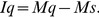
(1)


The focus on these competing motivations is not novel; other models of health behavior change, such as PMT [17] and the HBM [18], suggest that intending to quit an unhealthy behavior is a function of influences on these competing motivations. Indeed, any theory of rational choice suggests that all the smoker needs in order to quit is more desire to do so than to continue smoking [27].

The distinction between the motivations stems from the reward system’s powerful influence on goal seeking [21] and its circuits specialized for detecting both harmful (negative) and beneficial (positive) environments [28]. These circuits produce corresponding forms of negative and positive affect that, respectively, underlie desires to avoid or approach such objects as cigarettes [29]. Although these desires are often reciprocally related, they are independent sources of motivation that can have unique influences and effects.

In addition to the essential role of desires, the EDM recognizes that motivation is also determined by the perceived efficacy to satisfy desires. Self-efficacy is a familiar concept that has long been featured in models of behavior change [30]. In regard to smoking, even if smokers desire to quit the habit, they are unlikely to try unless they believe that they can implement the behavior. Neuroscience models of addiction also focus on self-efficacy by emphasizing the important role of the brain’s control system in undermining the ability to quit an addiction. Theories of behavior change, such as the TRA (15), treat desires (e.g., attitudes) and efficacy as additive influences on intentions. However, because both efficacy and desire are needed to motivate behavior, the EDM treats these expectancies and desires as multiplicative determiners of motivation, a common assumption in psychological models of motivation [31]. Thus, inserting the respective efficacies (*Eq, Es*) and desires (*Dq, Ds*) for quitting and smoking into eq. (1) produces:
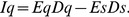
(2)


For an addiction such as smoking, both components of *Ms* are likely to be high. Indeed, the more one practices a behavior, the greater the skill and sense of efficacy for controlling it [32]. The same is unfortunately not the case for quitting. Even if the smoker wants to quit (*Dq*), no motivation and hence intention will be formed unless the smoker’s sense of efficacy (*Eq*) is also high. As is often the case for addictive habits [33], the smoker may strongly desire to quit but not believe that it is possible to do so. However, in deriving predictions from this model, it is important to consider individual differences in the various components of the model and the ways in which they are related to each other.

**Figure 1:**
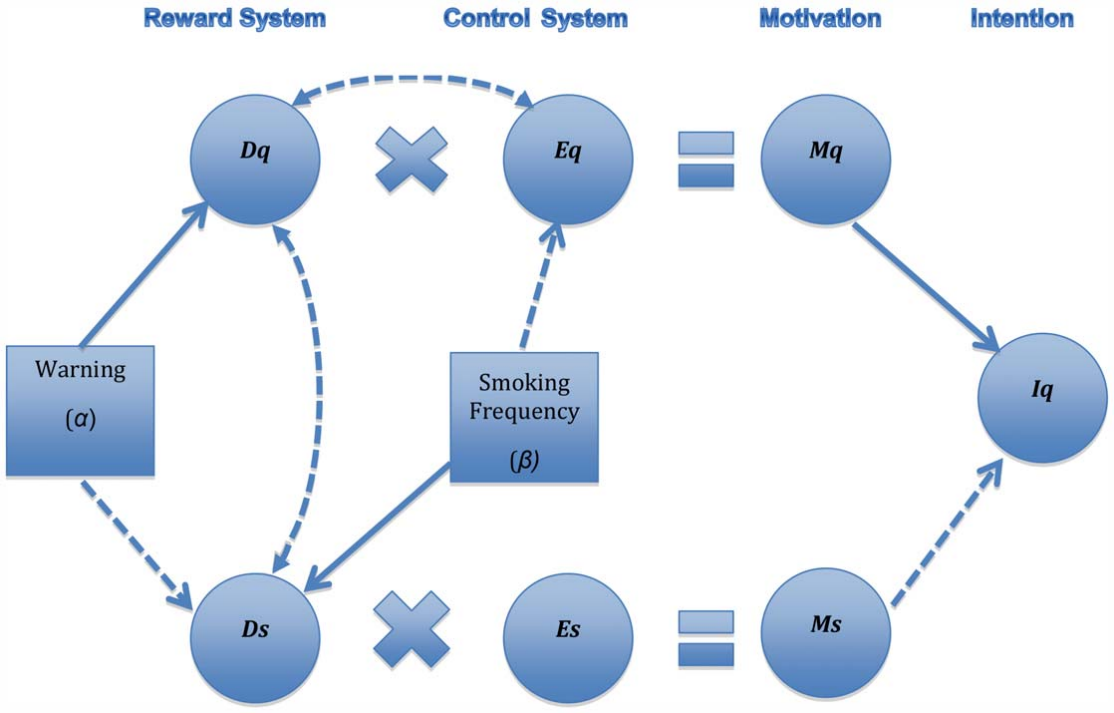
Efficacy-Desire Model of quit intentions showing relations between components of the model as they relate to the reward and control systems of addiction models. *Dq* and *Ds* are the desire to quit and smoke, respectively; *Eq* and *Es* are efficacies for quitting and smoking respectively. Respective interactions between efficacy and desire lead to *Mq* and *Ms*, which directly affect the intention to try quitting (*Iq*). Dashed paths indicate inverse relations; curved paths are correlations rather than effects.

Figure 1 shows the relations between the components of the model at the individual level. Although desire and efficacy to engage in a behavior are likely to be positively related, we show no relation between *Ds* and *Es* under the assumption that *Es* has reached asymptote in most smokers, leaving little room for any relation with *Ds*. However, for *quitting* an addictive habit, such as smoking, the relation between efficacy and desire to quit is likely to be negative. Efficacy for quitting the behavior is at its peak in the early stages of acquiring the habit, usually in adolescence, when the young smoker believes he or she will not have much difficulty stopping [34]. However, as the habit progresses, the smoker finds it increasingly difficult to stop even if the desire to do so increases. This process creates an inverse relation between *Eq* and *Dq*.

Neuroscience models of addiction specifically predict that greater frequency of smoking (represented in Figure 1 by parameter *β*) reduces *Eq* while it simultaneously increases *Ds.* It is also likely that frequency of smoking increases *Dq*, given what we know about smokers’ wishes to quit. But even leaving this out of the model, neuroscience models of addiction predict that the more one smokes, the lower one’s quit-efficacy (hence lower *Mq*) and the greater one’s desire to continue smoking (hence higher *Ms*). This disparity between *Mq* and *Ms* makes it difficult for the smoker to quit and shows why smokers are so conflicted by their addiction, wishing to quit but nevertheless continuing the habit.

The model makes interesting predictions regarding the effects of a warning, which, based on what we know about their effects [9]–[11], should increase *Dq* and reduce *Ds*. Figure 1 shows such a warning (whose intensity is indicated by parameter *α*) directly affecting *Dq*. Because *Dq* and *Eq* are inversely related, the effect on *Dq* is:
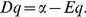
(3)


Replacing *Dq* in eq. (2) with eq. (3) shows that the model makes the novel prediction that for an addictive habit, *Mq* is an inverted U-shape function of *Eq*:
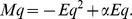



That is, assuming that individual differences in *Eq* range from negative to positive valence, *Mq* rises as *Eq* increases, but at an intermediate point, begins to decline. Although theories of behavior change predict that efforts to change behavior increase as efficacy increases, the EDM suggests that, for an addictive habit, this effect only holds up to a point, after which the motivation to do so declines. Furthermore, whether the habit is addictive or not, the effect of *α* depends on *Eq*, with an enhanced effect for positive values of *Eq* and a depressed effect for negative values of *Eq*.

The path linking *Ds* and *Dq* in Figure 1 suggests that these desires should be inversely related. However, consistent with a bivalent model of affect [29], we assume that these desires are somewhat independent. Hence, the effect of the warning on *Ds* is:
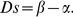
(4)


Eq. (4) expresses the intuitive result that *Ds* is positively related to the heaviness of the habit (*β*) and inversely related to the strength of the warning (*α*). Inserting eqs. (3) and (4) into eq. (2) yields the following overall relationship between *Iq* and the respective efficacies for quitting and smoking:
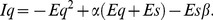
(5)


**Figure 2:**
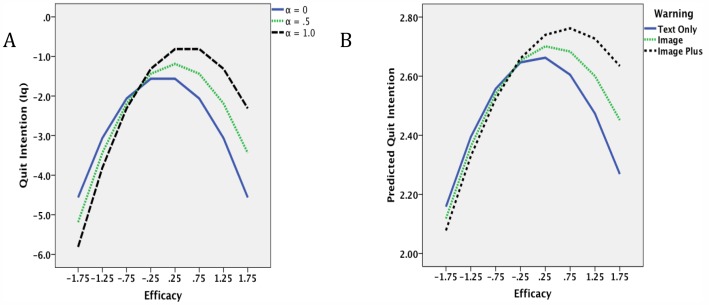
Relations between intention to quit smoking (*Iq*) and quit efficacy (*Eq*) by three levels of warning intensity (*α*) scaled from 0, .5. to 1.0. Panel A shows the relation using eq. (5) with *Es* fixed at.5 and *β* at 3.0. Panel B shows the observed relation using scores predicted by regression model in Table 4 (step 1).

We show examples of the hypothetical relation between *Iq* and *Eq* for different values of *α* in Figure 2A, assuming that *Es* and *β* are constant and that *Es* is higher on average than *Eq (.5 vs. 0).* The inverted U-shape relation is especially apparent when *α*  = 0. This shows that persons who smoke will have equally weak intentions to quit smoking not only when their efficacy is low but also when it is high. Indeed, absent any health warnings, smokers will have the greatest intentions to quit when their efficacy is at a moderate level. The prediction that low efficacy produces low intentions is not surprising since most theories expect this result. However, the model also predicts that those who think they can quit easily will not be motivated to do so either. This is a critical prediction of the model that will be tested for the first time in the present research.

A second critical prediction of the model is the effect of the warning. As seen in Figure 2A, the effect of *α* primarily increases the intention to quit among those with 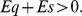 That is the point in the relation where all three curves in Figure 2A converge. Indeed, those with weaker *Eq* than −*Es* actually begin to exhibit a *reduction* in quit intention. Thus, the model makes the counterintuitive prediction that those with the strongest desires to quit (i.e., those who have smoked the longest) will be least motivated to respond rationally to warnings about the hazards of their habit, and this will be the case despite the fact that their response to the warning (created by an increase in *α*) is just as strong as the response among those with weaker desires to quit. This may explain the paradoxical effects of warnings observed in previous research. The quit-enhancing effects of increases in *α* will primarily be observed among those whose efficacy for quitting exceeds their efficacy for smoking. Indeed, warnings for those with weak efficacy for quitting will actually result in weaker intentions to quit.

We tested the major predictions of the model in an experimental context in which smokers were randomly assigned to see one example of a pack of cigarettes with a warning that was varied systematically in intensity across experimental conditions. This provided the opportunity to observe the effects of a warning in the context of individual differences in both the efficacy and desire components of the model. In addition to the predicted U-shape function shown in Figure 2A, we tested the prediction that increases in the intensity of warnings (represented by *α*) produced by adding an emotionally charged picture will lead to divergent effects on *Iq* depending on *Eq.* In addition, the EDM predicts that the greater the amount smoked (*β*), the lower the intention to quit. However, variation in *β* should only shift the curve up or down (it should be independent of *Eq* and *α*), and it should only interact with *Es*, which we assume is at a high and relatively fixed level for all smokers. In addition, as shown in Figure 1, frequency of smoking should be inversely related to *Eq* but positively related to *Ds*. Finally, in support of the expected inverse relation between *Eq* and *Dq*, length of time smoking (using age of the smoker as a proxy) should be positively related to *Dq* but negatively related to *Eq*.

## Methods

### Ethics Statement

The research was reviewed and approved by the Institutional Review Board (IRB) of the University of Pennsylvania, which adheres to the principles of the Belmont Report. As the survey was conducted over the Internet, was completed anonymously, and posed minimal risk, the IRB waived the requirement for written consent. However, participants were informed that the survey involved research, that their responses were anonymous, and that their participation was entirely voluntary. Thus, proceeding to take the survey was considered documentation of consent.

### Materials and Participants

We tested the model’s predictions using warnings that were tested for use in the U.S. by the FDA [13]. We used the same adult Internet panel used by the FDA (Research Now [35]) and similar warning labels that FDA evaluated. However, smokers who had participated in the earlier FDA test were excluded from the study.

**Figure 3:**
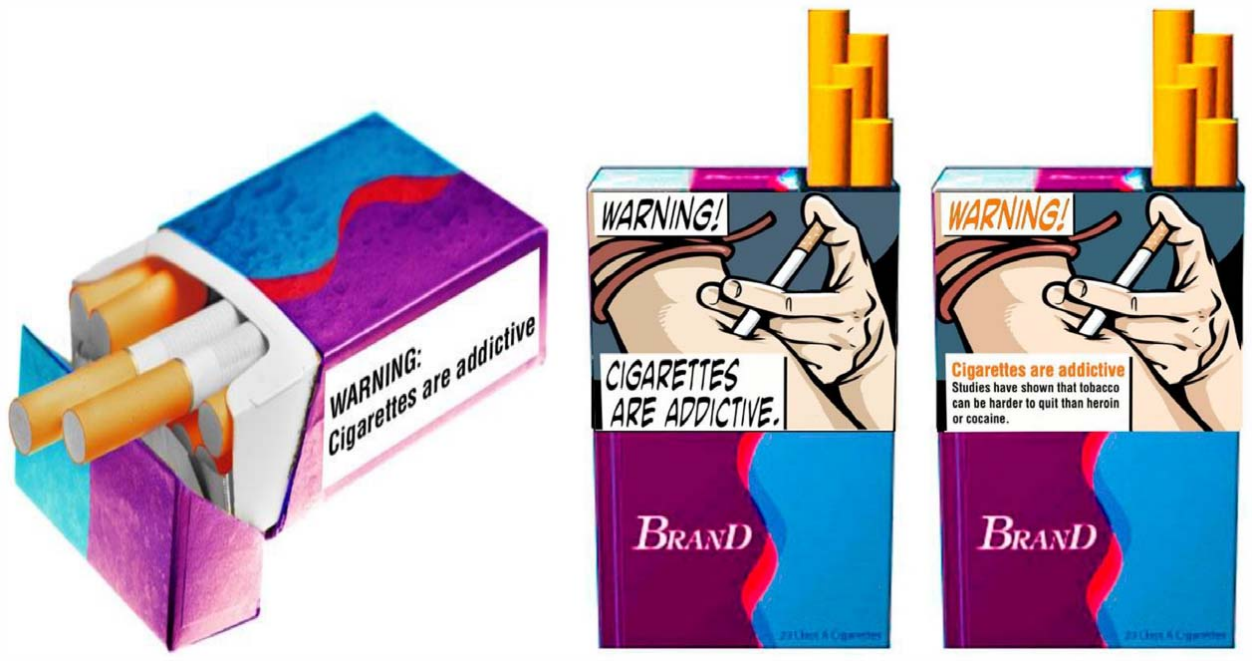
Examples of warning labels with (A) text on side of pack, (B) picture and text on front of pack, and (C) picture, text and elaboration on front of pack. Reprinted from www.fda.gov under a CC BY license, with permission from the FDA, copyright 02/24/2012.

Panel members were included in the study if they reported having smoked at least 100 cigarettes in their lives and if they currently smoked cigarettes “every day” or “some days” (N = 3297). Most of the sample reported smoking every day (62%), a lower proportion than the nearly 80% observed in recent national surveys of U. S. smokers [36]. In this test, approximately 160 smokers (56.5% female) in each of two age groups (18–24 and 25+ years) were randomly exposed to one of 10 computer screen images of a cigarette pack containing a warning about the hazards of smoking (see examples in Figure 3). The mean ages of the two age groups were 22.1 (SD  = 1.60) and 44.3 (SD  = 13.91). As expected, the older smokers were more likely to smoke every day compared to the younger group (76.5% vs. 45.7%), *X^2^*(1)  = 329, *p*<.001.

At the lowest level of intensity (*α*  = 0), the smoker saw a hypothetical pack of cigarettes on its side with one of 3 text warnings recently mandated by the U. S. Congress (Figure 3A): Cigarettes cause cancer, Cigarettes are addictive, or Smoking during pregnancy can harm your baby. These statements are factually correct but do not convey the importance and emotional impact imparted by pictorial warnings [9], [10]. In the middle level of intensity (*α*  = .5), the smoker saw the front of a similarly designed pack but with both the text and a picture that covered the top half of the pack (Figure 3B). In the third condition (*α*  = 1), the smoker saw a frontal view of the pack with a picture, the base text, and in addition, explanatory text that elaborated on the basis for the warning (Figure 3C). Elaborated warnings have been used in Canada since they were introduced in 2000 [9]. We expected the additional text to enhance the warning, resulting in the highest level of *α*. There were two versions of the elaborated text for the addiction and pregnancy warnings and one for the cancer warning, but we did not include a picture plus base text version for the cancer message. Thus, there were 10 different conditions with respondents nested within each condition in the experiment.

Respondents were permitted to view the warning image for as long as they wished; however, they were not permitted to return to it after leaving the screen. They then answered a series of questions about their reaction to the warning. We assessed quit intentions (*Iq*) with the following question: “How likely do you think it is that you will try to quit smoking within the next 30 days?” This question format is commonly used to determine intent because it captures both the desire and ability to engage in the behavior within a specified time period [15]. This question was answered using a scale from (1) very unlikely to (4) very likely. Those with no opinion (3.2%) were assigned the score of 2.5.

We assessed efficacy beliefs for quitting (*Eq*) by averaging agreement with two moderately correlated items (*r*  = .33, *p*<.001): “It is hard for a smoker to quit smoking” (reversed scored) and “I do not need help from anyone to quit smoking.” Both items were rated on a scale from (1) strongly disagree to (5) strongly agree. We also assessed previous quit attempts with the question: “During the past 12 months, have you stopped smoking for one day or longer because you were trying to quit smoking?” (Yes/No). This item was assessed to provide a behavorial measure of quitting that should also exhibit the inverted U-shape relation with *Eq* prior to exposure to the warning.

We assessed the direct effects of the warnings on measures of *Dq* and *Ds* as checks on the success of the manipulation of warning intensity (*α*). Our measure of desire to quit smoking (*Dq*) was response to: “How much do you want to quit smoking?” with answers ranging from “not at all” (1) to “a lot” (4). To assess *Ds*, we asked two questions that probed emotional reactions to smoking and desire to smoke a cigarette: “Imagine you are smoking right now. How good or bad would you feel smoking a cigarette right now?” with responses ranging from “very good” (1) to “very bad” (4). The other item asked for agreement with: “I want a cigarette right now” (reversed scored). The items were correlated (*r*  = .33, *p*<.001) and were averaged to define a measure of aversion to smoking, the inverse of *Ds*. As expected by the model in Figure 1, our measures of *Dq* and -*Ds* were correlated (*r*  = .25, *p*<.001). Finally, answers to the question about frequency of smoking, “Do you smoke, every day (1), some days (0) or never?” were used to assess *β*.

We used linear regression to test eq. (5) using self-efficacy to quit as a measure of *Eq* and three levels of *α* (0,.5, 1) as values representing the experimental warning conditions. A test of the effect of *β* was conducted with a second model in which smoking frequency was added as a predictor. Predicted scores from these models were plotted to provide a visual comparison of model predictions with those in Figure 2A. A test of the U-shape relation between *Eq* and prior quitting behavior was also tested using logistic regression and quit attempts in the 12 months prior to the experiment. We conducted regression analyses to test the predicted effects of age, *α* and *β* on all of the observed mediators in Figure 1 (*Eq, Dq, −Ds*). We did not have a measure of *Es*, which we assumed was relatively high for all smokers and which should be positively related to *β* in any case. A test of the critical hypothesis that efficacy for quitting is inversely related to desire to quit was conducted by regressing *Dq* on *Eq*. As predicted by eq. (3), those with the weakest efficacy should have the strongest desire to quit.

## Results

Table 1 provides the response distributions for the major variables in the analysis. To assess the success of randomization to conditions, we examined differences between conditions on several outcomes. There were no differences between the 10 conditions in efficacy for quitting, F(9,3287)  = .868, *p*  = .55; the proportion of respondents who smoked every day (vs. some days), *X* ^2^(9)  = 10.32, *p*  = .33; or who had tried to quit in the past 12 months, *X* ^2^(9)  = 9.41, *p*  = .40.

**Table 1:** Response distributions of major variables in the study (N  = 3297).

Variable	Frequency	%
**Quit Desire (** ***Dq*** **)**		
Not at all (1)	231	7.0
A little (2)	636	19.3
Somewhat (3)	1014	30.8
A lot (4)	1310	39.7
No opinion (missing)	106	3.2
**Quit Intention (** ***Iq*** **)**		
Very Unlikely (1)	736	27.3
Somewhat unlikely (2)	816	24.7
Don’t Know (2.5)	106	3.2
Somewhat likely (3)	822	24.9
Very likely (4)	817	24.8
**Aversion to Smoking (−** ***Ds*** **)**		
1.0 (Lowest)	110	3.3
1.5	191	5.8
2.0	504	15.3
2.5	786	23.8
3.0	823	25.0
3.5	606	18.4
4.0	206	6.2
4.5 (Highest)	71	2.2
**Quit Efficacy (** ***Eq*** **)**		
−1.75 (Lowest)	255	7.7
−1.25	501	15.2
−.75	693	21.0
−.25	661	20.0
.25	466	14.1
.75	216	6.6
1.25	252	7.6
1.75 (Highest)	253	7.7

**Figure 4:**
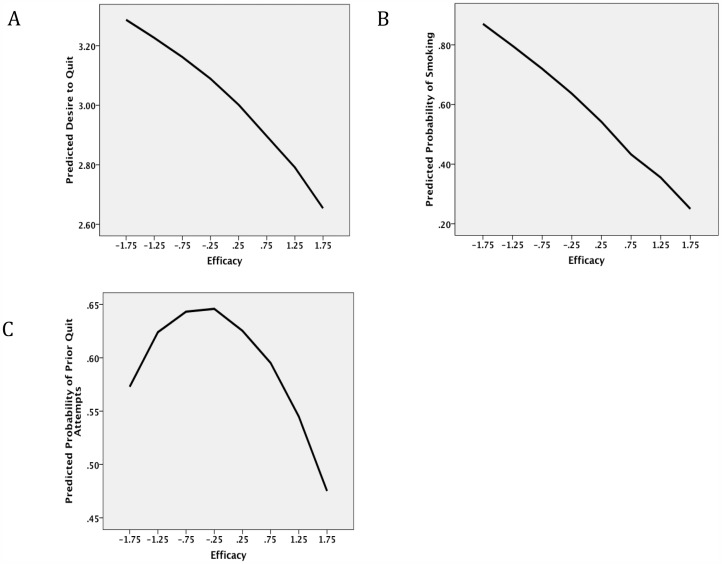
Predicted relations between three measures and efficacy to quit based on regression models in Tables 2 and 3: (A) desire to quit; (B) probability of smoking frequency; and (C) probability of trying to quit in past 12 months.

Tables 2 and 3 show the results of regression analyses relevant to the predictions in Figure 1. Consistent with eq. (3), *Eq* was inversely related to *Dq* (Table 2 and Figure 4A). This confirms the hypothesis that forms the basis for the U-shape relation between efficacy and intention to quit smoking. Related to this hypothesis is the prediction that smoking frequency (*β)* is inversely related to quit efficacy (*Eq*). As seen in Table 3 and Figure 4B, this hypothesis was supported, with each increasing unit on the efficacy scale associated with lower rates of daily smoking. Frequency of smoking was also inversely related to *−Ds*. Table 2 also shows the relation between age and the three outcomes, all of which are consistent with the model. Age was positively related to *Dq* and negatively related to *Eq.* It was also positively related to *−Ds*.

**Table 2:** Parameters of linear regression models for desire to quit smoking, aversion to smoking, and quit efficacy with warning and efficacy as predictors.

	Dependent Variable								
Predictor	Desire to Quit Smoking (*Dq*)			Aversion to Smoking (*−Ds)*			Quit Efficacy (*Eq*)		
	B	SE	Prob	B	SE	Prob	B	SE	Prob
Gender	.009	.034	.795	.085	.026	.001	−.078	.026	.002
Age Group	.147	.035	<.001	.105	.027	<.001	−.213	.027	<.001
Warning (α)	.026	.019	.174	.069	.015	<.001	−.002	.016	.915
Efficacy (*Eq*)	−.180	.018	<.001	.025	.014	.076			
Efficacy^2^ (*Eq* ^2^)	−.024	.016	.139	−.002	.012	.887			
Smoke Frequency (*β*)	−.118	.037	.002	−.439	.029	<.001	−.617	.027	<.001

Note: Males were coded as 0 and females as 1; Age was coded as 0 for 18–24 and 1 for 25+; Smoke frequency was coded 1 for daily smokers and 0 for less often. B is the unstandardized coefficient, SE is the standard error, and Prob is the probability of null hypothesis.

**Table 3:** Parameters of logistic regression models for relation between efficacy and frequency of smoking and prior attempts to quit.

	Dependent Variable					
Predictor	Frequency of Smoking (*β*)			Prior attempts to Quit		
	B	SE	Prob	B	SE	Prob
Gender	−.045	.081	.577	−.055	.076	.471
Age Group	1.17	.081	<.001	−.570	.080	<.001
Warning (α)	.015	.046	.739	.066	.042	.118
Efficacy (*Eq*)	−.764	.045	<.001	−.340	.041	<.001
Efficacy^2^ (*Eq* ^2^)	.137	.042	.001	−.148	.035	<.001
Smoke Frequency (*β)*				−.767	.087	<.001

Note: B is the unstandardized coefficient, SE is the standard error, and Prob is the probability of null hypothesis.

Smoking frequency was negatively related to *Dq* in Table 2. However, this could happen because it was positively related to *Ds*, which would counteract a potentially positive relation with *Dq*. To evaluate this possibility, we conducted a separate analysis in which *Ds* was held constant. This analysis confirmed that smoking frequency was positively related to *Dq* (B  = .134, se  = .037, *p*<.001), which would add further to the negative relation between *Eq* and *Dq*.

Examining the rate at which smokers had tried to quit in the past 12 months provided a test of the predicted inverted U-shape relation between *Eq* and behavior. As seen in Figure 4C, this relation exhibited an inverted-U shape as defined by the logistic regression model in Table 3. That model also found that smoking frequency was inversely related to prior quit attempts.

As expected, experimental variation in warning intensity (scaled 0, .5, 1) increased aversion to smoking (*−Ds*), one indicator of *α*. This effect was independent of efficacy, age, and smoking frequency, indicating that smokers across the efficacy continuum and ages recognized the implications of the warnings. It is noteworthy that *Eq* was unrelated to aversion to smoking. This was in contrast to its negative relation with desire to quit smoking. This difference is actually consistent with our predictions concerning each desire. It should be recalled that *Ds* was a function of *β* – *α,* while *Dq* was a function of *α – Eq.* Thus, one would not expect *Eq* to predict –*Ds,* holding constant *β*. It would nevertheless be expected that the warning would affect both desires. It is disappointing to find that the effect of *α* was not significant for our measure of *Dq*. A more sensitive measure of *Dq* may have allowed the relation between warning and *Dq* to emerge more clearly. Nevertheless, the measures of –*Ds* and *Dq* were related (*r*  = .25, *p*<.001), which supports their predicted relationship in the model.

Figure 2B shows the relation between *Iq* and *Eq* as a function of the three different levels of warning intensity as predicted by the regression model in Table 4. Comparing the result with the prediction in Figure 2A indicates a remarkably similar pattern to what the model predicted. First, the overall relation between intention and *Eq* exhibited the inverted-U shape. Second, the interaction between warning level and quit efficacy was significant: Intentions to quit smoking were elevated as a function of *α* primarily among those with efficacy scores above the estimated level of *Es*, which appeared to be approximately .40 in this sample (based on the intersection of the three curves in Figure 2B). The mean level of *Eq* in the sample was only −.22 (se  =  .017), considerably lower than the observed level of *Es* in Figure 2B. Thus, only those with scores of *Eq*>*−Es* (representing about 36% of the sample) were likely to exhibit greater intentions in response to the warning. The remaining 64% either did not change or became somewhat less likely to intend to quit. Finally and not surprisingly, the simple effect of *α* did not contribute to prediction. Thus, without examining the interaction between efficacy and warning level, one would conclude (as observed before) that warnings have little overall influence on intentions to quit.

**Table 4:** Regression parameters in test of Efficacy-Desire Model.

	Dependent Variable					
Predictor	Intention to Quit Smoking Step 1			Intention to Quit Smoking Step 2		
	B	SE	Prob	B	SE	Prob
Gender	.026	.034	.441	−.016	.038	.680
Age Group	.001	.034	.976	.028	.040	.475
Warning (α)	.019	.027	.476	.024	.030	.418
Efficacy (*Eq*)	−.031	.029	.276	−.040	.044	.371
Efficacy^2^ (*Eq* ^2^)	−.085	.027	.001	−.164	.040	<.001
α × *Eq*	.053	.019	.006	.066	.021	.002
α × *Eq* ^2^	.028	.018	.116	.016	.020	.437
Smoke Frequency (*β*)				−.460	.054	<.001
*β* × *Eq*				.007	.046	.883
*β* × *Eq* ^2^				.039	.041	.336

Note: B is the unstandardized coefficient, SE is the standard error, and Prob is the probability of null hypothesis.

To evaluate the success of the predicted relation between *Iq* and both *Eq* and *α*, we compared the variance explained by this model that only used 5 degrees of freedom to a model that included separate predictors for each of the 23 degrees of freedom represented by the fixed effects of the two predictors. Our model explained 71% of the fixed effects in the data despite using many fewer degrees of freedom. In addition, after accounting for the variation that was due to sampling error (which can be estimated by the within-subject mean square which was approximately 15% of the total), it is likely that the model accounted for over 80% of the reliable variation in the fixed effects.

**Figure 5:**
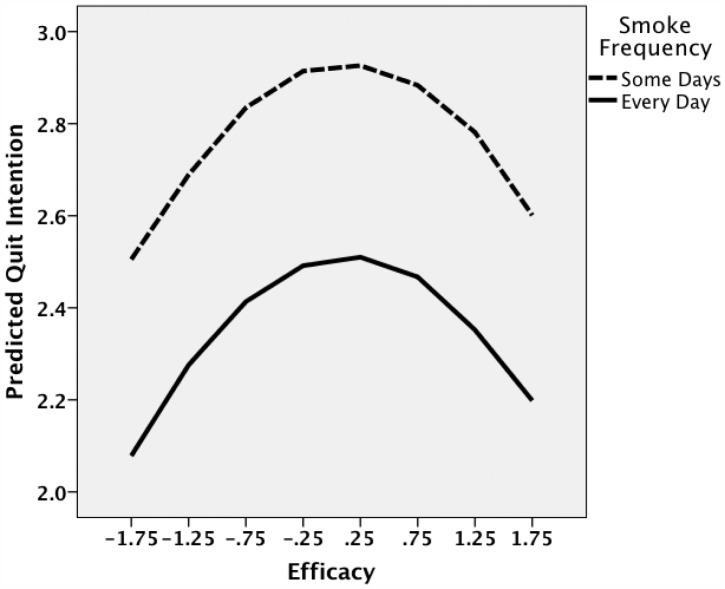
Predicted relation based on regression model in Table 4 (step 2) between intention and efficacy to quit with frequency of smoking as the parameter.

A final prediction of the model concerned the level of current smoking (*β*). We tested this effect by examining the relation between *Iq* and *Eq* for daily smokers versus those who smoked less often. As seen in Table 4 (step 2), smoking frequency predicted quit intentions apart from efficacy and warning intensity as predicted by eq. (5). Figure 5 shows that the curves predicted by the model in Table 4 conformed to prediction, with the curve shifting down for daily smokers without changing the inverted U-shape of the relation.

## Discussion

Our results showed strong support for predictions from the EDM. The inverted U-shape function between quit efficacy and strength of intentions to quit smoking was supported for both quit attempts in the past year and in response to a warning about cigarette hazards. This finding reflects the model’s unique prediction that given smoking’s addictive properties, the desire to quit smoking (*Dq*) and efficacy to do so (*Eq*) are inversely related and that these components of the decision model combine multiplicatively to form the motivation and hence intention to quit. The model’s prediction regarding the effect of a warning on quit intention was also supported in that the effect of the warning was only favorable for those with *Eq*>*−Es*. Indeed, as *Eq* declined, the warning became ineffective, exhibiting a boomerang effect. Finally, the prediction that smoking frequency would shift the intention curve up or down but would not interact with quit efficacy was also supported.

The findings show why previous research has failed to observe effects of warnings on the quit intentions of smokers at the population level [13], [14]. Even though smokers at all levels of efficacy recognized the implications of the warnings (as assessed by the effect on *−Ds*), it was primarily smokers with stronger quit-efficacy who reported increased intentions to try to quit. Ironically, the smokers who most desired to quit (i.e., those with lower efficacy) displayed reduced intentions. Thus, the effects of health warnings were limited to lighter smokers who have relatively stronger efficacy beliefs regarding quitting than they have regarding smoking. This pattern is consistent with research showing that it is primarily lighter smokers that are successful in quitting programs [37], [38]. Thus, the results suggest that the question regarding pictorial warnings is not whether they are effective in helping smokers to quit, but for which groups they are likely to be helpful. Our findings and the EDM indicate that level of smoking and efficacy for quitting are important parameters in determining the effects of pictorial warnings.

Although most theories of health behavior change would predict that smokers with greater quit efficacy would be more likely to respond favorably to a warning, the EDM is unique in predicting this outcome when *Eq*>*−Es.* It is also unique in its ability to predict the conditions under which one will observe a boomerang effect. Finally, the EDM uniquely predicts the inverted U-shape relation between *Eq* and *Iq* for an addictive habit. We thus see the model as providing important insights into the effects of health warnings that have not been predicted by previous theories of behavior change.

### Boomerang Effects of Health Messages

The finding that smokers with weaker self-efficacy for quitting actually became less intent on quitting following exposure to warnings is not a new phenomenon. This adverse effect of health information has been observed in studies of smoking and alcohol use [17]. Indeed, the classical study by Janis and Feshbach [39] found that, as the fear arousing character of messages increased, intention to follow through with the recommended health practice declined. This finding led to the prediction that fear arousing messages will exhibit an inverted U-shape relation to behavior change, whereby increases in fear initially lead to greater change but to decreases after an intermediate level has been reached. Contrary to this hypothesis, however, subsequent research failed to find the inverted U-shaped relation. Indeed, most research finds a weak but positive relation between fear arousal and message acceptance [40].

The EDM predicts a positive relation between the intensity of the message (i.e., *α*) and intentions/behavior when the efficacy for the recommended behavior exceeds that of the pre-existing unhealthy behavior. However, the model predicts *decreases* in behavioral intentions for those with relatively weak self-efficacy. It is quite likely that this was the case in Janis and Feshbach, which tested the effects of complex recommendations for repeated tooth brushing in early adolescents. Thus, the EDM could predict the effect observed by Janis and Feshbach and others that messages that increase fear regarding the recommended behavior will nevertheless backfire for those who have weak efficacy to change. The finding that such boomerang effects occur more often for addictive behaviors [17] is also consistent with the model. Furthermore, the EDM provides more precise predictions for the conditions under which one can expect such boomerang effects.

The tendency to reject messages as they increase in fear-arousing capacity has been ascribed to “defensive processing” in which the recipient of the message argues against the message and thus rejects its recommendation [40]–[42]. However, the EDM and the present results suggest that defensive processing is not necessary to explain message rejection. Smokers at all levels of self-efficacy reported increased unpleasant thoughts about smoking and felt disinclined to smoke a cigarette shortly following exposure to the warning. These reactions suggest that they did not reject the message outright. Instead, the results are more consistent with the prediction from the EDM that the multiplicative relation between desire and self-efficacy to quit leads to less motivation to adopt the recommended behavior among those with low *Eq*. As their desire to quit increased, their negative level of self-efficacy reduced rather than increased their motivation to quit, leaving them less intent on quitting.

### The Inverted U-Shape Relation

Aside from effects of warnings on quit intentions, the EDM predicts that addictive behaviors will exhibit an inverted U-shape function in relation to quit efficacy. This novel prediction was supported both for prior attempts to quit as well as immediate reactions to warnings. Thus, it was not just intentions that reflected this pattern but reports of behavior. Although this relationship has not to our knowledge been identified before, it is consistent with the observation that those who engage in behaviors that are difficult to quit often lose the motivation to desist from the unhealthy habit even if they succeed in reducing the behavior [33]. The EDM’s explanation for this phenomenon is that as the efficacy for quitting increases, it is matched by reduced desire to quit. As a result, the motivation to quit declines. However, increasing the desire to quit, as was done in the present experiment, should allow the beneficial effects of high quit efficacy to emerge.

The model also shows why smokers find it so difficult to quit. If smokers succeed in reducing their smoking habit as represented by the influence of *β* (perhaps by ingesting nicotine through a patch), they will experience less *Ds*, which will reduce *Ms*. Indeed, the model indicates that a reduction in *β* will raise *Iq* independent of *α* and *Eq*. However, as proposed in Figure 1, a reduction in *β* increases *Eq*. This moves them closer to the right side of the inverted-U relation with *Iq*. Thus, any favorable effects mediated by *Ds* will be offset and potentially outweighed by an opposite effect on *Eq*. These opposing forces could actually lead to a *decrease* in quit intention. As a result, once an addictive habit is created, the forces that motivate it can entrap the addict in a difficult to break cycle of continued dependence on the habit.

Despite the favorable effects of warnings suggested by the EDM, the model also predicts that the quit intentions of those with weak efficacy beliefs will be reduced as a result of exposure to warnings. This suggests that an effective public health strategy will require a two-pronged approach. One priority should be to encourage all smokers to reduce their habit as a transitory goal to eventually quitting. Although most smokers may not be able to quit, they may be able to reduce the strength of their habit. Indeed, recent surveys suggest that U.S. smokers have been doing exactly that [36]. The model suggests that recent efforts to reduce *Es*, through price increases and restrictions on smoking in public, may have been responsible for these reductions. That is, as long as 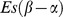 is greater than zero, decreasing *Es* should increase *Iq*. Since this condition is likely to be the case for heavy smokers, efforts to reduce *Es* should increase *Iq*. However, because reductions in smoking can also sap the motivation to quit, it will also be necessary to counteract this tendency with repeated exposure to health warnings that are periodically refreshed so that their effect does not wear off. This could enable persons who smoke to maintain stronger quit intentions and to break free of the habit.

### Limitations and Conclusions

This study has limitations that should be examined in subsequent research. The findings are based on only a single exposure and the effects of warnings may intensify as smokers are exposed to warnings over time. The model predicts that the lightest smokers with the strongest self-efficacy for quitting will be most successful in quitting. However, we have not tested this hypothesis here. We also did not have a very sensitive measure of *β*, which the model suggests is a major factor in intentions to quit. Finally, we did not assess self-efficacy for smoking, which the model predicts will have an influence on quitting. We assumed that it was relatively high compared to self-efficacy for quitting. However, future research should look at variation in this form of self-efficacy as well. This parameter could be assessed by asking about barriers to smoking that the smoker experiences, such as restrictions on places to smoke, increases in prices for cigarettes, and social disapproval for smoking.

In conclusion, the EDM sheds light on why models that assume a rational response to warnings do not account for the behavior of persons addicted to a behavior such as smoking. The model translates insights from current neurobiological models of addiction [19], [22]–[25] into concepts that have been employed in models of behavior change [15], [17], [18]: namely, attitudes toward addictive habits (desire) and the lack of control over their cessation (self-efficacy). The model is also consistent with neuroscience theories that postulate separate approach and avoidance motives that underlie affective experience and that can produce extreme conflict in persons with a serious addiction such as smoking. Thus, the model shows how the barriers to quitting an addictive habit such as smoking are so imposing that they can trap the person who is addicted in a continuing cycle of dependence and frustrate efforts to reduce this life-threatening habit.
